# *Ewingella Americana*: An Emerging True Pathogen

**DOI:** 10.1155/2012/730720

**Published:** 2012-06-14

**Authors:** Syed Hassan, Syed Amer, Chetan Mittal, Rishi Sharma

**Affiliations:** Division of Internal Medicine, Henry Ford Hospital, Detroit, MI 48202, USA

## Abstract

Infections caused by *Ewingella americana* have been rarely reported in the literature. Most of the cases that have been reported were among the immunocompromised patients. We report a case of *E. americana* causing osteomyelitis and septic arthritis of the shoulder joint in a previous intravenous drug abuser. The causative pathogen was identified by synovial fluid analysis and culture.

## 1. Introduction

*Ewingella americana* is a rare gram negative, lactose fermenting, oxidase negative, catalase positive, indole negative, facultative anaerobic bacillus first described from clinical specimens in 1983 by Grimont et al. [1], as a new group in the Enterobacteriaceae family. It rarely causes human infections and has been identified from various clinical samples including sputum [2], conjunctiva [3, 4], blood [5–8], wound [9], and peritoneal dialysate [10]. Interestingly, it has also been isolated from the intestinal contents of snails and slugs [11], fresh nutria carcasses [12], vacuum packaged meat [13], and mushrooms [14] as well.

This case report is the first clinical description of *E. americana* causing osteomyelitis and septic arthritis of the shoulder joint.

## 2. Case Report 

**Figure 1:**
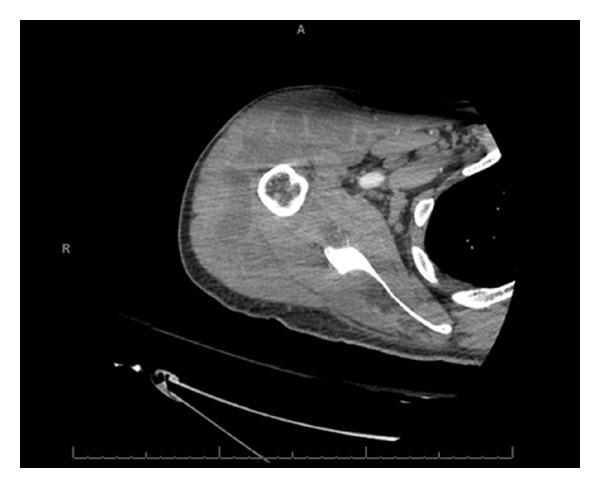
In the right upper extremity, there is a full thickness rotator cuff tear with a glenohumeral joint effusion and subacromial subdeltoid bursal fluid. Pockets of gas are noted in the glenohumeral joint effusion and the bursal fluid, suggestive of septic joint and septic bursitis with intramuscular abscesses.

A 50-year-old male was admitted to the hospital, with gradual onset of pain and swelling in the right shoulder since 4 days. He denied fever, chills, or rigors. His past medical history was significant for hypertension and intravenous drug abuse. His last use of heroin was 2 months ago with an unsterilized needle contaminated by saliva, in the right arm. Physical examination revealed limited range of motion of right shoulder secondary to pain. Radiograph of the right shoulder was suggestive of osteomyelitis. A computed topographic scan of the shoulder revealed multifocal, intraarticular abscess formation involving the right upper extremity. It also showed erosion of the humeral head and the glenohumeral joint, consistent with septic arthritis (Figure 1). Arthrocentesis was done, and the cell count of the synovial fluid showed white blood cell count of 9.4 × 10^9^/L (90% neutrophils, 1% bands). Cultures of the synovial fluid grew *Ewingella americana*. The antimicrobial susceptibility test carried out by disk diffusion method showed susceptibility to amikacin, ampicillin, cefazolin, gentamicin, piperacillin tazobactam, tobramycin, and trimethoprim sulfamethoxazole while was resistant to ciprofloxacin.

The patient was started on ceftriaxone 2 gm intravenous every 24 hours. He continued to improve and was discharged home to complete 6 weeks of intravenous antibiotic therapy. He was followed 4 weeks later in the outpatient clinic and showed resolution of infection which was confirmed both clinically and by imaging. The antibiotic course was completed with no complications.

## 3. Discussion

Clinical infections due to *E. americana* have been reported to cause peritonitis [10], conjunctivitis [3, 4], bacteremia [8], and pneumonia [2, 15]. Colonization in wound [9] and sputum [2] were also reported in patients without causing clinical infection. Sepsis [5–8] and even death from Waterhouse-Friderichsen syndrome due to *E. Americana* [16] has also been reported.

*E. Americana* is seen in patients who were immunosuppressed due to diabetes mellitus [5], bone marrow transplantation, chemotherapy [7], end stage renal disease [10], and use of mercaptopurine [15]. Although, a few cases of *E. americana* have been reported earlier causing conjunctivitis [3, 4] and Waterhouse-Friderichsen syndrome [16] in previously healthy individuals, this is the first case of osteomyelitis involving the joint due to intravenous drug abuse. Based on this observation, clinicians may want to consider *Ewingella Americana* as an emerging true pathogen. 

Since little information exists on the ecological niche of this organism, in our case we speculate on the source of contamination as saliva from the patient's mouth. Kati et al. [10] proposed domestic water to be a source of infection in peritonitis of a patient undergoing peritoneal dialysis. A contaminated ice bath was identified as the probable source in an outbreak of *E. americana* bacteremia, in patients who had undergone cardiovascular or peripheral vascular surgery [8]. Maertens et al. [7] speculated on inadequate hand hygiene as the source of infection. *E. americana* is an organism without nutritional needs that can survive in water and citrate solution and preferably grows at 4°C. 

The only risk factor in our patient was that he was an intravenous drug abuser while using unsterilized needles for his heroin injections; he might have inoculated the pathogen into his blood causing transient blood stream infection which ultimately seeded into his shoulder joint.

To our knowledge, this case is the first report of osteomyelitis and septic arthritis caused by *E. americana* infection. Earlier reports suggested *E. americana* causing infections in immunocompetent host; however, in our case the infections were certainly favored by intravenous injections with contaminated material.
